# Microfoamed Strands by 3D Foam Printing

**DOI:** 10.3390/polym14153214

**Published:** 2022-08-07

**Authors:** Daniele Tammaro, Massimiliano Maria Villone, Pier Luca Maffettone

**Affiliations:** Dipartimento di Ingegneria Chimica, dei Materiali e della Produzione Industriale, University of Naples Federico II, P.le Tecchio 80, I-80125 Napoli, Italy

**Keywords:** 3D printing, polylactic acid, microfoamed strand, CO_2_, foaming, porous structures, fusion deposition melting, 3D foam printing

## Abstract

We report the design, production, and characterization of microfoamed strands by means of a green and sustainable technology that makes use of CO_2_ to create ad-hoc innovative bubble morphologies. 3D foam-printing technology has been recently developed; thus, the foaming mechanism in the printer nozzle is not yet fully understood and controlled. We study the effects of the operating parameters of the 3D foam-printing process to control and optimize CO_2_ utilization through a maximization of the foaming efficiency. The strands’ mechanical properties were measured as a function of the foam density and explained by means of an innovative model that takes into consideration the polymer’s crystallinity content. The innovative microfoamed morphologies were produced using a bio-based and compostable polymer as well as polylactic acid and were then blown with CO_2_. The results of the extensive experimental campaigns show insightful maps of the bubble size, density, and crystallinity as a function of the process parameters, i.e., the CO_2_ concentration and temperature. A CO_2_ content of 15 wt% enables the acquirement of an incredibly low foam density of 40 kg/m^3^ and porosities from the macro-scale (100–900 μm) to the micro-scale (1–10 μm), depending on the temperature. The foam crystallinity content varied from 5% (using a low concentration of CO_2_) to 45% (using a high concentration of CO_2_). Indeed, we determined that the crystallinity content changes linearly with the CO_2_ concentration. In turn, the foamed strand’s elastic modulus is strongly affected by the crystallinity content. Hence, a corrected Egli’s equation was proposed to fit the strand mechanical properties as a function of foam density.

## 1. Introduction

Polymeric foams are widely used in several technological fields (e.g., the biomedical, aerospace, nautical, sport, and leisure fields), offering distinctive features that derive from their porous internal morphology [1]. The characteristic size of the pores, their shape, and their hierarchical organization are important factors in determining the structure–property relationship in these materials. For instance, hierarchical porous structures outperform their non-hierarchical counterparts with respect to mechanical behavior and accessible active surface [2,3]. As a matter of fact, nature has often chosen optimized hierarchical foams to shape life on our planet. Many examples are present in nature where high performances are reached at a minimum material cost using hierarchically structured foams [4]. Natural cellular materials, such as bamboo and beeswax honeycomb [5], usually have complex hierarchical geometries, designed to carry out a specific task or optimize a specific property. As an example, cellular structures in nature have a high stiffness-to-weight ratio, high crash energy absorption, high fire resistance, non-flammability, non-toxicity, low thermal conductivity, magnetic permeability, and a lower density than its counterparts without pores. In human bones, the fine design of the cell orientation and size is used to obtain excellent mechanical properties with the lowest possible weight. In butterfly wings, a foamed hierarchical structure optimizes the energy reflection at a minimum weight cost. Scientists have often taken inspiration from natural cellular materials for engineering applications, particularly with respect to light weight construction, crash energy absorption, noise control, heat exchange, purification, decoration and arts, and sound damping [6,7,8].

Recently, many efforts have been made to 3D print polymers into hierarchical foams or mesh structures by piling up extruded strands, where macroscale and microscale pores are generated by the computer-designed spacing between the filamentary struts [9,10]. In these cases, the extruded strands are still dense, and the inter-strand porosity is directly restricted by the printing resolution. Some attempts have been made to produce 3D-printed foams with both high inter-strand and intra-strand porosities, in which each single printed strand is foamed [11]. The current solutions are based on a two-stage approach: in the first step, the structures are printed with inter-strand porosity, then the intra-strand porosity is produced by freeze-drying or batch-foaming [12]. Recently, a solution has been proposed where inter- and intra-strand porosities are produced in one step by means of an inline or discontinuous solubilization of a physical blowing agent [2]. However, those approaches do not enable the fine control of the foamed-strand morphologies and limitations exist in designing innovative morphologies [13]. A smart design and control of 3D foam printing that can address such limitations and cover a wide range of controlled pore sizes and morphologies is highly demanded to enable the full exploitation of the rich design space offered by hierarchical structures.

In this work, we report the design, production, and characterization of microfoamed strands made by means of a green and sustainable technology that uses carbon dioxide, CO_2_, to create ad-hoc bubble morphologies. As the result of an extensive experimental campaign, we present insightful maps of bubble morphology as a function of the process parameters, namely, the temperature and CO_2_ concentration. The innovative microfoam morphologies were obtained using a biodegradable and bio-based polymer such as polylactic acid, PLA, blown with CO_2_, thus allowing for a sustainable production process. The increase of the CO_2_ content to 15 wt% enables the attainment of an incredibly low density of 40 kg/m^3^ and a micrometric pore size. The foam’s crystallinity content changes linearly with the CO_2_ concentration up to 15 wt%. In the literature, it is known that the crystallinity level affects the elastic modulus of semicrystalline polymers. Herein, a corrected Egli’s equation is proposed to model the elastic modulus of the microfoamed strands as a function of the expansion ratio (i.e., at different densities), taking into account—for the first time—the effect of the strands’ crystallinity. The proposed approach opens the way to a broad scenario of opportunities to design the mechanical, thermal, and electrical properties of 3D foam printed structures.

## 2. Materials and Methods

Polylactic acid (PLA), grade NW2003D, was supplied by NatureWorks LLC (Plymouth, MN, USA) and its physical properties, whose values are taken from the technical data sheet of the material, are summarized in Table 1.

PLA pellets were dried overnight at 60 °C in oven under vacuum conditions before any manipulation. The drying protocol was based on literature data that report an efficient removal of humidity after 12 h at 60 °C [14]. A PLA filament with diameter of 1.75 mm was produced using a Composer350 extruder (from 3DEVO company, Utrecht, The Netherlands), whose process parameters are given in Table 2. A Prusa Research I3 Mk3s 3D printer was then used to foam the filament. The blowing agent, CO_2_, was supplied by Aircos (Napoli, Italy).

The foams were characterized to determine their bulk density (*ρ*) and cell density (*N*). The *ρ* was measured according to ASTM D7710 by using an analytical balance (Mettler Toledo, Columbus, OH). The samples were first sectioned with a razor blade in liquid nitrogen and then coated with gold through a sputter coater. The cellular structures of the foams were investigated by a scanning electron microscope (Hitachi TM 3000 SEM). The SEM images reported in the manuscript refer to cross-sections randomly chosen from the foamed samples. Each foaming experiment was repeated at least three times to calculate the cell size distribution $N=E{\left(n/A\right)}^{3/2}$, where E is the expansion ratio and *n* is the number of cells in the area *A* of the SEM micrograph. The cell size was measured according to ASTM D3576. The experimental expansion ratio was calculated as $E=\rho_{p}/\rho$, where $\rho_{p}$ is the density of the solid sample. The foaming efficiency was calculated as the ratio between the experimental and the theoretical foam density, i.e., the density that would be obtained if all the blowing agent contained in the polymer was entrapped in the final foam.

**Figure 1:**
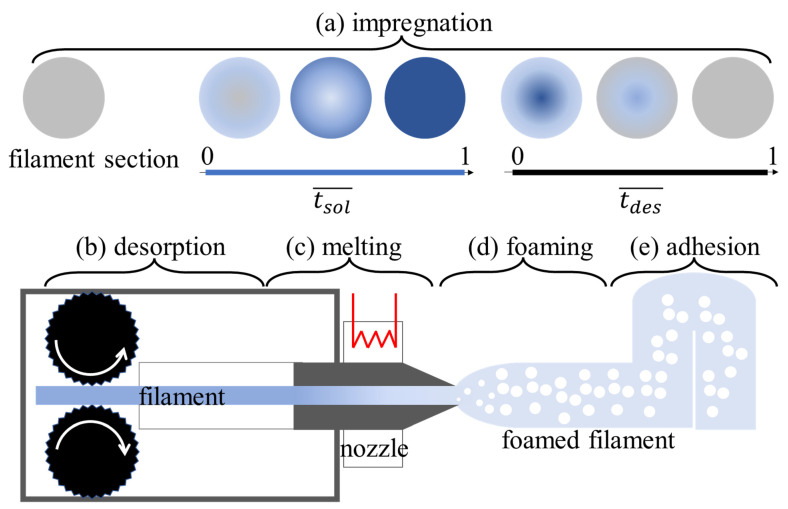
Schematic representation of the 5 steps to print foamed strands: (**a**) impregnation, (**b**) desorption, (**c**) melting, (**d**) foaming and (**e**) adhesion among the strands.

The process used for producing the microfoamed strands consists of the following steps: (1) solubilization of the blowing agent in the filament to be foamed, (2) diffusion of the blowing agent throughout the filament, (3) foaming at the exit of the printer nozzle, (4) stabilization, and (5) deposition of the foamed filament. Hence, the proposed technology can be schematized with the following units: (a) impregnation of the filament with the blowing agent, (b) desorption of the blowing agent, (c) melting, (d) foaming, and (e) adhesion of the strand (see Figure 1). Some more details on units a–d are given below.

(a) Solubilization: The thermoplastic polymeric filament is impregnated within a high-pressure and high-temperature autoclave. In this work, we used CO_2_ as the blowing agent for the impregnation of PLA filaments.
(b) Desorption: When the filament exits the autoclave, the desorption starts. Here, a non-uniform blowing agent profile can be promoted within the filament, which in turn enables the acquirement of custom cellular morphologies in the foaming zone.
(c) Melting: As in the standard 3D printing process, the impregnated filament is melted before accessing the nozzle. Here, the gas solubilized into the filament may affect the melting kinetics and form bubbles if the pressure is not sufficiently high.
(d) Printing and foaming: The rapid pressure drop in the nozzle triggers bubble nucleation followed by bubble growth. Consequently, the printed strand reaches the desired density and morphology.

The solubilization and foaming processes involved in 3D printing are described in greater detail by Tammaro et al. [2]. It is worth noting that there are two important differences between this work and Tammaro et al.’s: (a) in this work, the sorption and desorption occur offline, so they are decoupled by the printing speed of the machine; (b) in this work, the use of an eco-friendly blowing agent, CO_2_, which is a gas at room temperature and pressure, requires the introduction of a high-pressure vessel for its solubilization. The filament impregnation depicted in Figure 1a can be described by the dimensionless solubilization and desorption times ${\bar{t}}_{\mathrm{sol}}=t_{\mathrm{sol}}/\left(R^{2}/D\right)$ and ${\bar{t}}_{\mathrm{des}}=t_{\mathrm{des}}/\left(R^{2}/D\right)$, where $t_{\mathrm{sol}}$ and $t_{\mathrm{des}}$ are the dimensional sorption and desorption times of the blowing agent, *R* is the radius of the filament, and *D* is the diffusivity of the blowing agent, equal to about $10^{-9}{\;\mathrm{cm}}^{2}/s$. For ${\bar{t}}_{\mathrm{sol}}<1$, we expect a concentration gradient across the filament section, and the larger ${\bar{t}}_{\mathrm{sol}}$ the larger the average concentration of the blowing agent within the filament. On the other hand, the blowing agent entirely impregnates the filament section when ${\bar{t}}_{\mathrm{sol}}>1$. In this work, we investigate filaments solubilized with ${\bar{t}}_{\mathrm{sol}}>1$ and ${\bar{t}}_{\mathrm{des}}<1$.

The accessible ranges of the operating conditions are reported by Tammaro et al. [2]. The nozzle temperature can be changed arbitrarily through the 3D printer software; conceptually, the lower limit is given by the melting temperature of the polymer (about 170 °C for PLA) and the upper limit is the temperature at which the polymer starts to degrade (about 280 °C for PLA) [14]. Hence, an experimental operating temperature range in between these two limits is considered. The software fixes the wall temperature of the brass nozzle in the 3D printer, which affects the melting kinetics of the filament and thus the instant at which the gas foaming starts. The foaming temperature, *T_foam_*, is defined to equate to the nozzle temperature. In addition, the printing speed can be changed at will by the 3D printer software, which sets the polymer flow rate through the nozzle. The largest speed is limited by slippage at the gears [15].

## 3. Results

In this section, we study the effects of the melt temperature and blowing agent concentration on the microfoamed strands.

The printing speed was fixed to 15 mm/s to have a melt with a homogenous temperature at the nozzle exit. Depending on the printing speed, there could be a strong temperature inhomogeneity in the polymer melt during the fusion deposition melting (FDM) of the thermoplastic filaments, which is due to the fact that the heating occurs via conduction with the metal wall [16]. The evaluation of the Graetz number, defined as the ratio between the heat diffusion characteristic time, $\tau_{\mathrm{diff}}$, and the characteristic residence time inside the nozzle, $\tau_{\mathrm{res}}$, helps to predict the temperature inhomogeneity of the FDM. Assuming perfect contact with the metal wall of the printer heater, $\tau_{\mathrm{diff}}$ can be calculated as $\tau_{\mathrm{diff}}=r_{\mathrm{heater}}^{2}/\alpha =3.5\;s$, where *r*_heater_ is the heater radius and $\alpha =K/\rho c_{p}$ is the thermal diffusivity, with *K* and *c*_p_ being the thermal conductivity and the specific heat of the polymer, respectively. Assuming a plug flow, $\tau_{\mathrm{res}}$ can be calculated as $\tau_{\mathrm{res}}=l_{\mathrm{heater}}/v_{\mathrm{in}},$ where $l_{\mathrm{heater}}$ is the heater length and $v_{\mathrm{in}}\;$ is the printing speed. In our experiment, $\tau_{\mathrm{diff}}\sim 3-4\;s$ and $\tau_{\mathrm{res}}$ is always above 10 s. Therefore, a temperature homogeneity is expected because $\tau_{\mathrm{res}}\gg \tau_{\mathrm{diff}}$. When the printing speed has a $\tau_{\mathrm{res}}<\tau_{\mathrm{diff}}$, the melt temperature will be inhomogeneous, and a different foaming process can be achieved. The 3D foam printing at a high speed will be investigated in detail in our future work.

**Figure 2:**
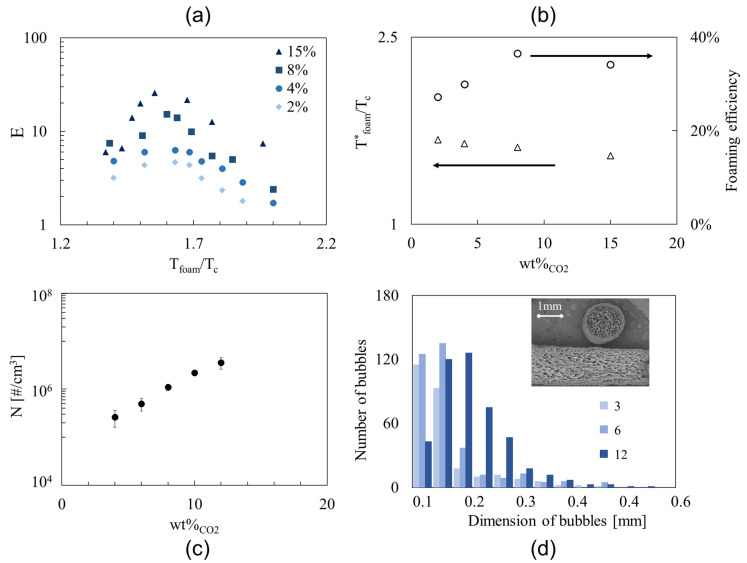
(**a**) Effects of foaming temperature and CO_2_ concentration on foam density; (**b**) Effects of CO_2_ concentration on the foaming temperature maximizing the expansion ratio (triangles) and on the foaming efficiency (circles); (**c**) Effect of CO_2_ concentration on bubble density at $T_{\mathrm{foam}}^{*}/T_{c}$; (**d**) Bubble size distribution for three foamed strands obtained at *T*_foam_ = 1.7*T*_c_ and 3, 6, and 12 wt%_CO_2__ (see legend). Insert: SEM picture of the cross and longitudinal sections of a foamed strand at 6 wt%_CO_2__.

The effect of the temperature on the foaming process is shown in Figure 2a, where the expansion ratio is plotted as a function of *T*_foam_/*T*_c_ for four values of the CO_2_ weight percentage. The crystallization temperature (*T*_c_) is assumed to be independent of the blowing agent concentration and equal to 120 °C [17]. We found that the expansion ratio trends have a bell shape, with a maximum corresponding to a value $T_{\mathrm{foam}}^{*}/T_{c}$ that decreases as the gas concentration increases, ranging from about 1.7 at 2 wt% to about 1.5 at 15 wt%. On the right side of the bell-shaped trends, the foaming of the strands is limited by the short time for the diffusion of the blowing agent from the surface compared to the crystallization time for setting. On the left side, the foaming of the strands is limited by the long viscous time for the bubble growth compared to the crystallization time for setting. The highest expansion ratio (i.e., 35) is achieved at the largest gas concentration, 15 wt%; interestingly, it is comparable with the expansion ratio that can be achieved in industrial extrusion foam processes [18]. One could imagine that since the microfoamed strands have a larger surface-to-volume ratio than the macroscopic industrial profiles (usually, on the centimeter-scale), this would lead to a faster escape of the blowing agent (the diffusion time depends quadratically on the radius of the strand) and correspondingly, to a worsening of the final foam morphology and density. However, especially at large amounts of blowing agent, the cooling time due to the fast adiabatic expansion (on the order of milliseconds) is always much lower than the diffusion time of the blowing agent in the printed strand (in the order of seconds); thus, it is possible to tune the operating parameters to avoid a worsening of the final foam density.

The trends of $T_{\mathrm{foam}}^{*}/T_{c}$ and of the foaming efficiency at $T_{\mathrm{foam}}^{*}/T_{c}$ as a function of the CO_2_ concentration are shown in Figure 1b. The optimal foaming temperature maximizing the expansion ratio decreases monotonically with the CO_2_ concentration (see the empty triangles in Figure 1b) because the plasticization effects enhance with the CO_2_ concentration [18]. The foaming efficiency reaches its maximum at 8 wt% of CO_2_, where the phenomena of the bubbles’ coalescence and gas escape are likely minimized (see the empty circles in Figure 1b). Our results are in good agreement with those by Mihai et al. [19], who found that in foam extrusion, a high concentration of CO_2_ enables the acquirement of closed-cell morphologies, taking advantage of the plasticization effect and the fast adiabatic expansion that stabilize the foamed structure.

The bubble density of the foamed strands at $T_{\mathrm{foam}}^{*}/T_{c}$ increases exponentially as a function of the CO_2_ concentration within the investigated concentration range, as reported in Figure 1c. This result is in good agreement with the prediction of the Classical Nucleation Theory, which assumes that no bubble coalescence or collapse occurs during the foaming process [20,21,22]. The bubble density in the microfoamed strands with CO_2_ was higher than that obtained by Tammaro et al. [2] by using acetone. Figure 1d provides the bubble size distributions of the samples obtained at $T_{\mathrm{foam}}=1.7T_{c}$ and three values of the CO_2_ weight percentage, i.e., 3, 6, and 12%, showing that it is possible to control the bubble size in foamed strands by simply tuning the CO_2_ concentration. The bubble size distribution is similar to that obtained by Tammaro et al. [2] using acetone as the blowing agent.

In conclusion, we can remark that at a low printing speed, despite the small characteristic size of the nozzle compared to a standard foam extruder, the cell size and foam density can be tuned by using the same leverages that are usually used in foam extrusion, namely, the melt temperature and the blowing agent concentration [23,24,25,26,27,28].

**Figure 3:**
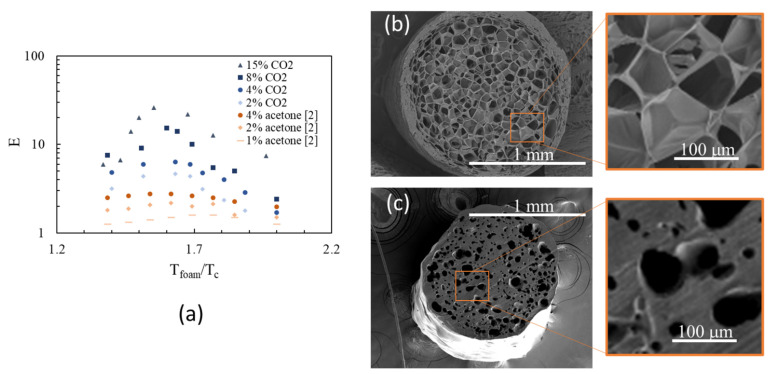
(**a**) Comparison between the expansion ratios achieved with CO_2_ (this work) and acetone [2]. SEM images of the cross section of a strand foamed with CO_2_ (**b**) and acetone (**c**).

A quantitative comparison between the outcomes of this work and those of a 3D foam-printing process with an inline solubilization [2] is shown in Figure 3a, reporting the expansion ratio as a function of $T_{\mathrm{foam}}/T_{c}$ for both cases. Bell-shaped curves are found for both the blowing agents, that is, CO_2_ and acetone. Considering the samples with the same solubilized blowing agent concentration (i.e., 4 wt%, see the blue and red dots), the maximum expansion ratio is about eight with CO_2_ and only about two with acetone. We speculate that the lower density achieved with CO_2_ is due to the plasticization effect that affects the crystallization rate of the PLA [29] and thus the ability to keep the gas after the bubble nucleation phase. Moreover, a qualitative comparison between the cross sections of the strands foamed with CO_2_ (Figure 3b) and acetone (Figure 3c) is presented. The sample foamed with CO_2_ shows a cellular morphology with trapezoidal bubbles that have grown until impingement [30], namely, when they interact with each other and are separated by thin polymer walls with a thickness of tens of microns. The polymer walls are almost all closed and just a few holes can be observed (see the magnification on the right of Figure 3b). With CO_2_, the thin polymer walls have been stabilized by some setting mechanism, such as crystallization, that avoids the rupture and the coalescence of bubbles (which usually results in an increase of the final foam density). The sample foamed with acetone (from Reference [2]) shows a cellular morphology that has not reached the impingement stage and some bubbles have likely undergone early coalescence [31], as suggested by the absence of thin polymer walls among them (see the magnification on the right of Figure 3c), pointing out the absence of any setting mechanism. The analysis of the cellular morphologies seems to confirm our speculation on the fact that CO_2_ enhances the polymer crystallization rate due to its plasticization effect [32], thus stabilizing the foaming process, increasing its efficiency, enabling the achievement of the impingement stage, and consequently a lower final foam density.

**Figure 4:**
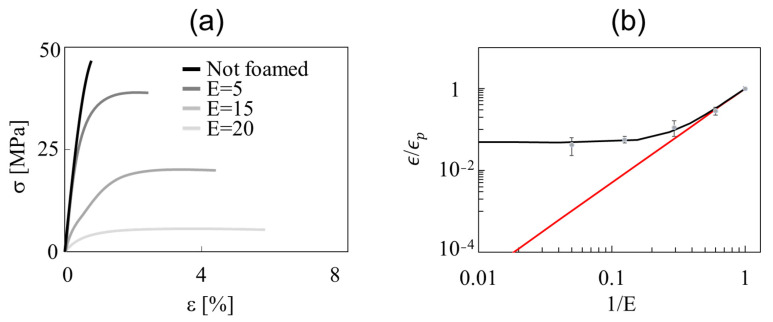
(**a**) Tensile stress as a function of percent deformation for 4 foamed strands at increasing *E* (see legend); (**b**) Ratio of foam and polymer elastic moduli as a function of the inverse of the expansion ratio. The red line is the best fit of the data at high 1/*E* through the power law proposed by Egli et al. [4]; the black curve is the best fit of the data through Equation (1).

The foamed strands were characterized mechanically by means of microtensile tests over a range of expansion ratios from 1 to 20. The entire stress (*σ*)-strain (ε) curves are shown in Figure 4a and the specific elastic modulus $\epsilon /\epsilon_{p}$, where ϵ and $\epsilon_{p}$ are the elastic moduli of the foam and the “pure” polymer, respectively, is reported in Figure 4b as a function of 1/*E*. In Figure 4a, it is evident that as *E* increases, the elongation at the break of the strands increases and the elastic modulus (i.e., the initial slope of the stress–strain curve) decreases. The specific elastic modulus decreases in turn with an increasing *E* (decreasing 1/*E*) (see Figure 4b). A simple power-law model was proposed by Egli to describe such a dependency: $\frac{\epsilon }{\epsilon_{p}}={\left(\rho_{p}/\rho \right)}^{n}={\left(1/E\right)}^{n}$. The exponent *n* was found to be close to two by many authors [24]. For our foamed strands at a high 1/E, where the power-law seems to hold, we found that the best fit of the experimental data yields *n =* 2.3 (see the red line in Figure 4b).

**Figure 5:**
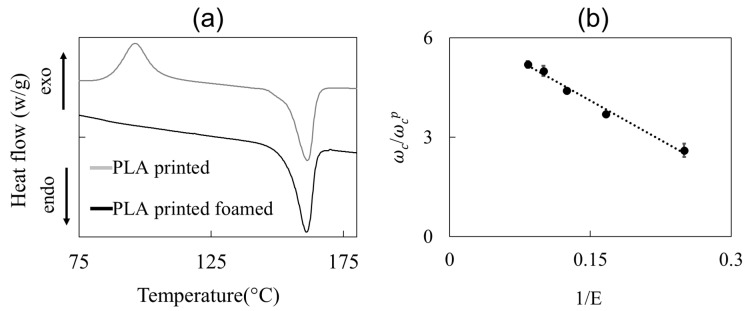
(**a**) DSC curves for a printed and foamed/printed PLA strand; (**b**) effect of expansion ratio on the crystallinity content—the dashed line is a guide for the eye.

The larger elastic modulus of the foamed PLA strands at a low density can be ascribed to the increase in the crystallinity due to (a) the larger plasticization effect at larger a concentration of the blowing agent and (b) the larger deformation rate due to the larger foam expansion [25]. The DSC measurements confirm that the crystallinity content increases as the density of the foamed strands decreases. In Figure 5a, the DSC scans of a non-foamed (grey curve) and a foamed (black curve) printed strand are shown. During the first heating ramp, a cold crystallization, i.e., the exothermic peak at ca. 110 °C, happens in the non-foamed strand, whereas the foamed strand has no cold crystallization due to the high level of crystallinity reached during the foaming process. The ratio $\omega_{c}/\omega_{c}^{p}$, where $\omega_{c}$ is the degree of crystallinity of the strand and $\omega_{c}^{p}$ that of the polymer, is plotted in Figure 5b as a function of the inverse of the expansion ratio, showing a linear monotonic decrease. Therefore, the data can be fitted with the law $\omega_{c}/\omega_{c}^{p}=\left(A-B/E\right)$, whose best-fit parameters are *A* = 0.26 and *B* = 0.22, yielding the dashed line in Figure 5b.

In the literature, it is known that the crystallinity content of foamed strands affects their mechanical properties [22]. This might be taken into account in the simplest way by adding a term to the rhs of Egli’s law, as follows:

(1) $$\frac{\epsilon }{\epsilon_{p}}={\left(\frac{1}{E}\right)}^{n}+\left(\frac{\omega_{c}}{\omega_{c}^{p}}\right)$$

By recalling that $\omega_{c}/\omega_{c}^{p}=\left(A-B/E\right)$, the “modified” Egli’s law finally reads

(2) $$\frac{\epsilon }{\epsilon_{p}}={\left(\frac{1}{E}\right)}^{n}+\left(A-\frac{B}{E}\right)$$

Such an empirical equation, with *n* = 2.3, *A* = 0.26, and *B* = 0.22 (see above), can satisfactorily describe our experimental data, as shown by the black line in Figure 3b (the adjusted coefficient of determination is equal to 0.98). Therefore, it enables the design of the mechanical properties of foamed printed strands in a wide range of densities, taking into account the important effects of the crystallinity on the elastic modulus, as reported by Frunzaverde et al. [26]. It is worth noting that the model works “well” because the dimensions and the orientation of the cells are similar among the samples at different densities. In future work, the production of printed structures with a larger difference in cell size and orientation will be tackled to investigate the effects found by Bao et al. on macroscopic foams [27]. On the other hand, it must be remarked that the extrapolation of Equation (2) at a very low 1/*E*, i.e., at a very large expansion ratio, might be “dangerous”, since it predicts $\epsilon /\epsilon_{p}\to A$, which does not make much sense when the polymer content inside a strand becomes negligible compared to the gas content. The novel microfoamed strands presented and discussed in this work can be used to produce a broad window of hierarchically structured porous materials, taking advantages of all the derived properties. For instance, they can open a new path to manufacture scaffolds for bone regeneration, where the optimization of the mechanical properties can be achieved with the model developed in this work.

## 4. Conclusions

We report the design of microfoam strands with complex foamed morphologies that have been produced by a cost-effective 3D foam printing technology. The results of the extensive experimental campaigns on polylactic acid blown with CO_2_ show the dependency of the foam density, bubble density, and crystallinity on the temperature and gas concentration. A CO_2_ content of 15 wt% enables the acquirement of an incredibly low density of 40 kg/m^3^ and a pore size on the microscale. Our results confirm that the main setting mechanism of foamed PLA strands is the adiabatic cooling due to the fast adiabatic expansion; hence, it is possible to tune the operating parameters to avoid a worsening of the final foam density. The foam crystallinity content changes linearly with the CO_2_ concentration up to 15 wt% and it has a strong effect on the strand elastic modulus. Based on our experimental data, we modified Egli’s law by considering an additive linear contribution of the crystallinity content to the elastic modulus.

## Figures and Tables

**Table 1:** Physical properties of PLA used to produce the filaments.

Physical Properties	Value	ASTM Method
Specific Gravity [g/cc]	1.24	D792
MFR [g/10 min] @ 210 °C/2.16 kg	6	
Relative Viscosity @ 1.0 g/dL in chloroform at 30 °C	4.0	D1238
Clarity	Transparent	D5225
Peak Melt Temperature [°C]	145–160	
Glass Transition Temperature [°C]	55–60	D3418
Tensile Yield Strength [MPa]	60	
Tensile Modulus [MPa]	3.6	D3418
Tensile Strength at Break [MPa]	60	D882

**Table 2:** Filament extrusion process conditions.

Screw Speed	Zone 1 (Feeding)	Zone 2 (Melting)	Zone 3 (Mixing)	Zone 4 (Shaping)	Fan Speed
5.0 rpm	150 °C	180 °C	200 °C	190 °C	500 rpm

## Data Availability

Not applicable.
